# Mathematical Model of Viral Kinetics In Vitro Estimates the Number of E2-CD81 Complexes Necessary for Hepatitis C Virus Entry

**DOI:** 10.1371/journal.pcbi.1002307

**Published:** 2011-12-08

**Authors:** Pranesh Padmanabhan, Narendra M. Dixit

**Affiliations:** 1 Department of Chemical Engineering, Indian Institute of Science, Bangalore, India; 2 Bioinformatics Centre, Indian Institute of Science, Bangalore, India; ETH, Switzerland

## Abstract

Interaction between the hepatitis C virus (HCV) envelope protein E2 and the host receptor CD81 is essential for HCV entry into target cells. The number of E2-CD81 complexes necessary for HCV entry has remained difficult to estimate experimentally. Using the recently developed cell culture systems that allow persistent HCV infection in vitro, the dependence of HCV entry and kinetics on CD81 expression has been measured. We reasoned that analysis of the latter experiments using a mathematical model of viral kinetics may yield estimates of the number of E2-CD81 complexes necessary for HCV entry. Here, we constructed a mathematical model of HCV viral kinetics in vitro, in which we accounted explicitly for the dependence of HCV entry on CD81 expression. Model predictions of viral kinetics are in quantitative agreement with experimental observations. Specifically, our model predicts triphasic viral kinetics in vitro, where the first phase is characterized by cell proliferation, the second by the infection of susceptible cells and the third by the growth of cells refractory to infection. By fitting model predictions to the above data, we were able to estimate the threshold number of E2-CD81 complexes necessary for HCV entry into human hepatoma-derived cells. We found that depending on the E2-CD81 binding affinity, between 1 and 13 E2-CD81 complexes are necessary for HCV entry. With this estimate, our model captured data from independent experiments that employed different HCV clones and cells with distinct CD81 expression levels, indicating that the estimate is robust. Our study thus quantifies the molecular requirements of HCV entry and suggests guidelines for intervention strategies that target the E2-CD81 interaction. Further, our model presents a framework for quantitative analyses of cell culture studies now extensively employed to investigate HCV infection.

## Author Summary

The interaction between the hepatitis C virus (HCV) envelope protein E2 and the host cell surface receptor CD81 is critical for HCV entry into hepatocytes and presents a promising drug and vaccine target. Yet, the number of E2-CD81 complexes that must be formed between a virus and a target cell to enable viral entry remains unknown. Direct observation of the E2-CD81 complexes preceding viral entry has not been possible. We constructed a mathematical model of HCV viral kinetics in vitro and using it to analyze data from recent cell culture studies obtained estimates of the threshold number of E2-CD81 complexes necessary for HCV entry. We found that depending on the E2-CD81 binding affinity, between 1 and 13 complexes are necessary for HCV entry into human hepatoma-derived cells. Our study thus presents new, quantitative insights into the molecular requirements of HCV entry, which may serve as a guideline for intervention strategies targeting the E2-CD81 interaction. Further, our study shows that HCV viral kinetics in vitro can be described using a mathematical model, thus facilitating quantitative analyses of the wealth of data now emanating from cell culture studies of HCV infection.

## Introduction

HCV entry into target cells is a complex process involving the interactions of the viral envelope proteins E1 and E2 and several cell surface receptors, namely, scavenger receptor class B type I (SR-BI) [1], the tetraspanin CD81 [2], [3], and the tight junction proteins claudin-1 (CLDN1) [4] and occludin [5]. Several recent studies suggest a central role for CD81 in HCV entry: E2 has been shown to interact directly with SR-BI and CD81 following viral attachment to a target cell [1], [2]. Patient derived neutralizing antibodies appear to target the CD81 binding domains on E2 [6]. Indeed, anti-CD81 antibodies were able to block infection in vitro [3] and in a mouse model [7]. Graft reinfection following liver transplantation was observed recently to select for HCV strains capable of more efficient entry, achieved partly through mutations in the CD81 binding domains on E2 [8]. Expression of human CD81 and occludin was essential for infection of genetically humanized mice [7]. Besides, CLDN1 appears to mediate HCV entry through its association with CD81 [9], [10]. Consequently, the E2-CD81 interaction presents a potent target for intervention; drugs that block the E2-CD81 interaction are currently under development [11], [12].

How many E2-CD81 complexes must be formed between a virion and a target cell to enable HCV entry? Knowledge of this threshold would determine the number of E2-CD81 complexes that a drug or a vaccine must prevent from forming in order to block viral entry, thus presenting a quantitative guideline for intervention strategies targeting the E2-CD81 interaction. This threshold is currently unknown. Direct observation of the number of E2-CD81 complexes formed before HCV entry has not been possible. Recent cell culture studies have determined the dependence of viral entry and kinetics in vitro on the CD81 expression level on target cells [10], [13]–[18]. In particular, cells expressing higher levels of CD81 were found to be more susceptible to infection [13]. Further, the frequency of cells with low CD81 expression typically increased with time following the exposure of cells to HCV [14], [15]. We reasoned that analysis of these observations using a mathematical model of viral kinetics, akin to studies of HIV entry (for example, see [19], [20]), may allow estimation of the threshold number of E2-CD81 complexes necessary for HCV entry. While models of HCV viral kinetics in vivo have been employed successfully to analyse patient data and elucidate guidelines for treatment [21]–[31], models of HCV viral kinetics in vitro have just begun to be formulated.

Here, we constructed a mathematical model of HCV viral kinetics in vitro that mimics cell culture studies of the dependence of viral entry and kinetics on CD81 expression. Model predictions captured data from several independent experiments quantitatively and yielded estimates of the threshold number of E2-CD81 complexes necessary for HCV entry.

## Results

### Model formulation

**Figure 1:**
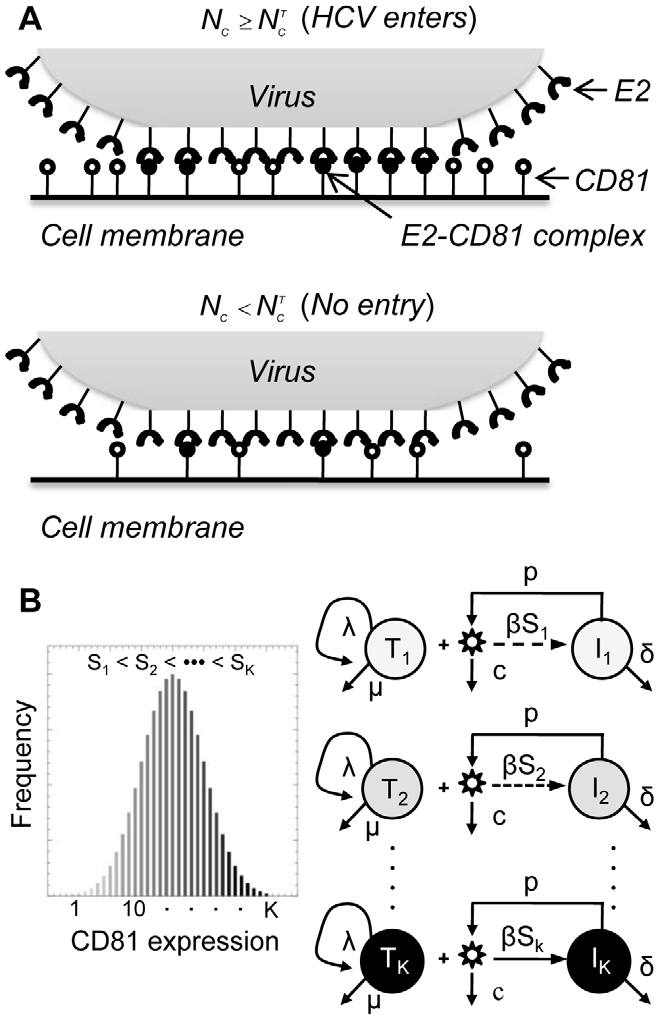
Schematic of the model. (A) HCV enters a target cell if the number of E2-CD81 complexes formed across the virus-cell pair, 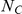, exceeds the threshold, 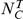 (top), but not otherwise (bottom). (B) Viral kinetics following the exposure of cells with a distribution of the CD81 expression level (left) to virions in vitro is determined by the time-evolution of subpopulations 1 through *K* of cells with distinct CD81 expression levels and hence different susceptibilities to HCV entry (right).

We considered in vitro experiments where a population of target cells, 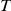, with a known distribution of the CD81 expression level across cells is exposed to a population of HCVcc (cell culture adapted) virions, 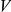, and the progression of infection followed [13]–[15]. We modelled the ensuing viral kinetics as follows (Fig. 1). We first considered a single virus-cell pair with the virus attached to the cell by interactions that precede E2-CD81 binding [12]. E2 and CD81 then interact to form E2-CD81 complexes. We computed the mean number of these complexes formed at equilibrium, 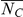, as a function of the CD81 expression level, 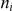, on the cell. Assuming that the number of complexes formed, 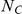, followed a Poisson distribution with mean 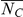, we computed the probability, 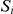, that 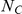 was larger than a threshold number 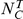. We assumed that viral entry (and subsequently infection) occurred if 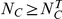 (Fig. 1A). Thus, 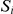 yielded the relative susceptibility to infection of a cell with CD81 expression level 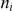.

We next considered the population of cells exposed to virions (Fig. 1B). We divided the cells into different subpopulations 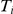 with distinct CD81 expression levels 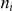 and hence different susceptibilities 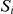, where 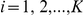. Cells in each subpopulation were assumed to proliferate, die, or be infected at a rate proportional to 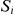. The resulting infected cells, 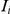, were lost at enhanced rates compared to 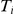 due to virus-induced cytopathicity in vitro [15], [32]. Free virions were produced by infected cells and were cleared. With this description, we constructed dynamical equations to predict the time-evolution of each of the uninfected and infected cell subpopulations and the population of free virions and compared our predictions with experiments (Methods).

### Model predictions

#### Triphasic viral kinetics in vitro

**Figure 2:**
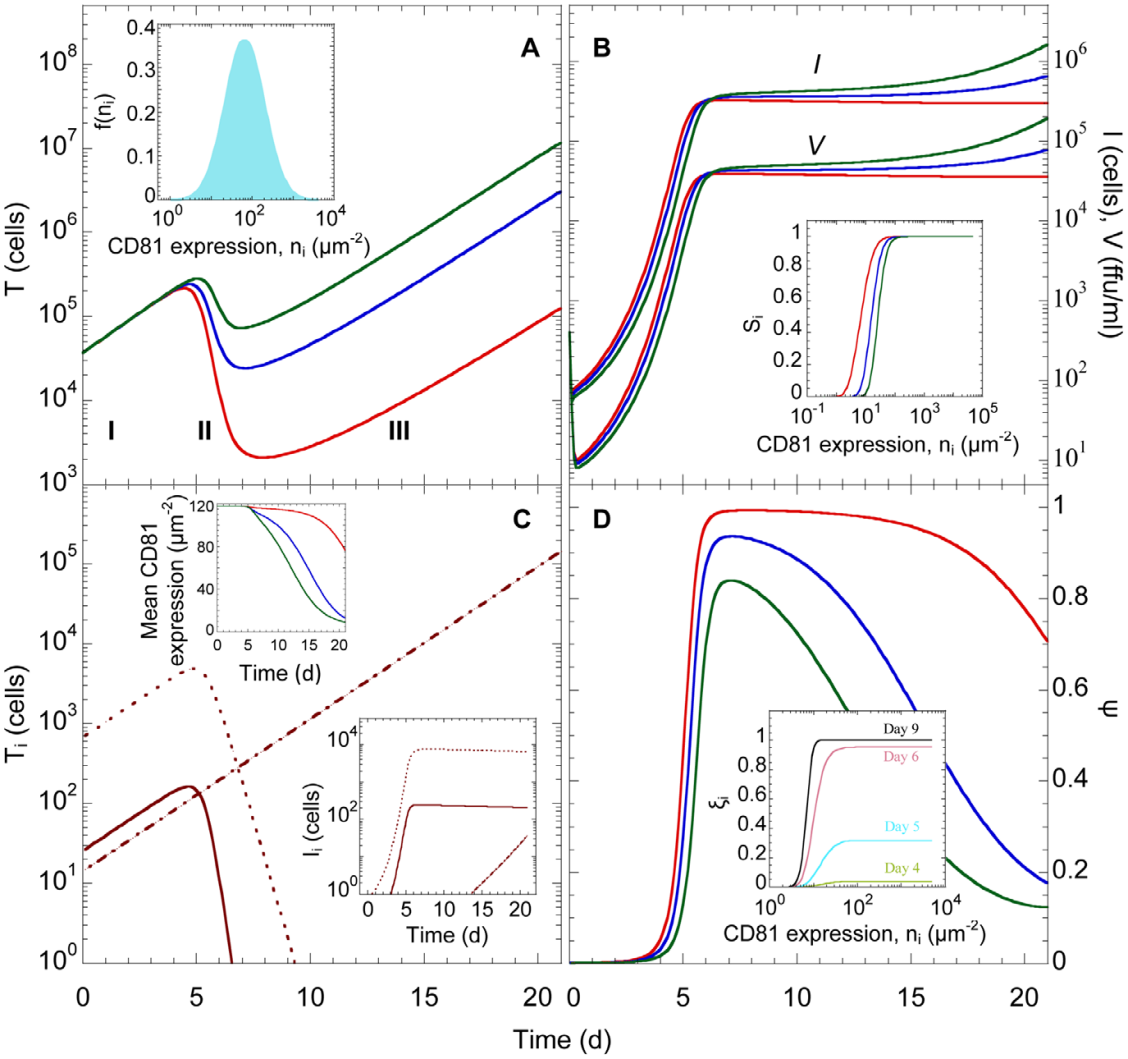
Model predictions of HCV viral kinetics in vitro. The time evolution of (A) uninfected cells, *T*, (B) infected cells, *I*, and viral load, *V*, (C) uninfected cell sub-populations, *T_i_*, and (lower inset) infected cell sub-populations, *I_i_*, corresponding to 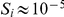 (dot-dashed line), 0.5 (dotted line) and 1 (solid line), and (D) the total fraction of cells infected, *ψ*. The initial CD81 distribution, 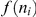, as a function of the CD81 expression level, 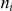, is presented in the inset in (A). The three phases of infection are also marked in (A). Inset in (B) shows 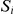 as a function of 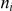. Upper inset in (C) shows the time evolution of the mean CD81 surface density. Inset in (D) shows the fractions of cells infected in sub-populations with distinct CD81 expression levels, *ξ_i_*, at different post-infection times. In (A), (B), (D) and the upper inset in (C), 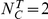 (red line), 4 (blue line), and 6 (green line). In (C) and the inset in (D), 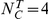. Other parameter values employed are 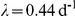; 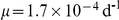; 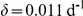; 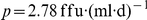; 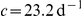; 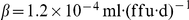;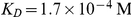. Initial conditions are 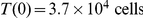; 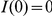; 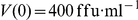.

When cells with a log-normal distribution of the CD81 expression level (Fig. 2A inset) were exposed to virions in vitro, we found that infection proceeded in three phases (Figs. 2A and B). In the first phase, the population of uninfected cells, 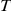, rose, as their net proliferation rate dominated their loss rate by infection. At the same time, exposure of 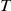 to virions resulted in the growth of infected cells, 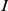. Initially, because 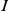 was small, viral production was dominated by clearance, resulting in a decline of the viral load, 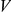. As 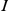 increased, viral production compensated for clearance and 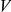 began to rise. Subsequently, 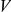 evolved proportionally to 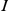, indicating the establishment of a pseudo-steady state between viral production and clearance, 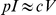.

In the second phase, 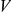 was sufficiently large that the loss of 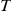 by infection outweighed cell proliferation, resulting in a decline of 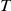 (Fig. 2A). This decline was consequently associated with a steep rise in 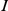 and correspondingly 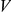 (Fig. 2B). In the third phase, 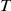 rose again while 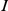 and 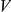 remained nearly constant. This third phase resulted from diverse kinetic profiles of subpopulations of cells, 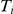, expressing different levels of CD81, which we describe below.

#### Dependence of viral kinetics on CD81 expression

The relative susceptibility of cells to infection, 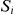, depends on the CD81 expression level, 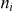, and the parameters 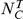 and 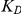 (equilibrium dissociation constant or the inverse of affinity) (Eqs. (4) and (5)). For fixed 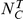 and 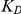, 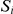 increased with 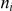 (Fig. 2B inset). Above a certain 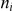 (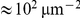), CD81 did not limit entry and cells were nearly completely susceptible to infection (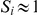). At the same time, below a certain 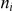 (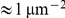), cells were refractory to infection (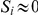). This variation in 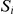 led to diverse kinetic profiles.

In the first phase of viral kinetics, all subpopulations 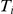 grew because infection rates were low and cell proliferation dominated (Fig. 2C). In the second phase, 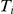 declined for subpopulations with sufficiently large 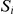 that infection dominated proliferation (e.g., 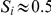 and 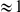 in Fig. 2C). The corresponding 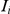 accordingly rose (Fig. 2C lower inset). Eventually, nearly all cells in these subpopulations were infected and 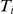 vanished leaving behind a pool of infected cells. This pool remained constant over the 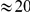 d period considered here because the lifespan of infected cells assumed was much larger, 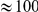 d. Correspondingly, 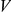 also remained nearly constant (Fig. 2B). In contrast, subpopulations with low 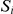 were not easily infected and continued to grow throughout (*e.g.*, 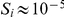 in Fig. 2C). The growth of the latter cells resulted in the rise of 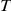 in the third phase of infection (Fig. 2A).

Indeed, at any time 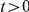, the fraction of cells infected within any subpopulation, 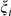, increased with 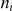 (Fig. 2D inset). For large 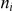, CD81 did not limit infection (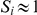) so that 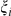 evolved independently of 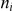. Eventually, all cells in the latter subpopulations were infected and 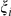 approached 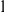. Below a certain 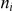, however, 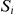 was so low that hardly any cells were infected and 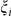 remained vanishingly small. These latter subpopulations, refractory to infection, ultimately dominated the target cell population. That a majority of the cells in the third phase was refractory to infection was also evident from the time-evolution of the total fraction of cells infected, 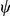 (Fig. 2D). 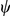 increased initially as cells with high 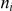 were infected. After a majority of the latter cells was infected, 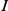 remained constant whereas the population of cells refractory to infection increased, resulting in a decrease of 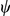. Accordingly, the mean CD81 expression also decreased (Fig. 2C upper inset). Even with low susceptibility, however, stochastic events may result in the formation of the threshold number of complexes and allow viral entry. As 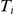 increased, the frequency of such stochastic events grew and resulted in the growth of the corresponding 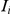 (Fig. 2C lower inset), which in turn resulted in the subtle rise of 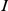 and 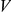 towards the end of the third phase (Fig. 2B).

#### Influence of model parameters

As 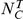 increased, 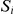 decreased (Fig. 2B inset), increasing the fraction of cells refractory to infection and hence lowering 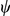 (Fig. 2D). Accordingly, the rise of 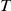 in the third phase was enhanced upon increasing 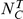 (Fig. 2A). As 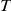 increased, the frequency of stochastic events leading to entry into cells with low 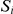 increased and resulted in a slightly greater increase of 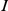 and 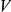 towards the end of the third phase (Fig. 2B). Variations in other model parameters introduced similar quantitative variations but did not alter the triphasic kinetics qualitatively (Fig. S1). Below, we present comparisons of our model with experiments.

### Comparison with experiments

#### HCV viral kinetics in vitro

**Figure 3:**
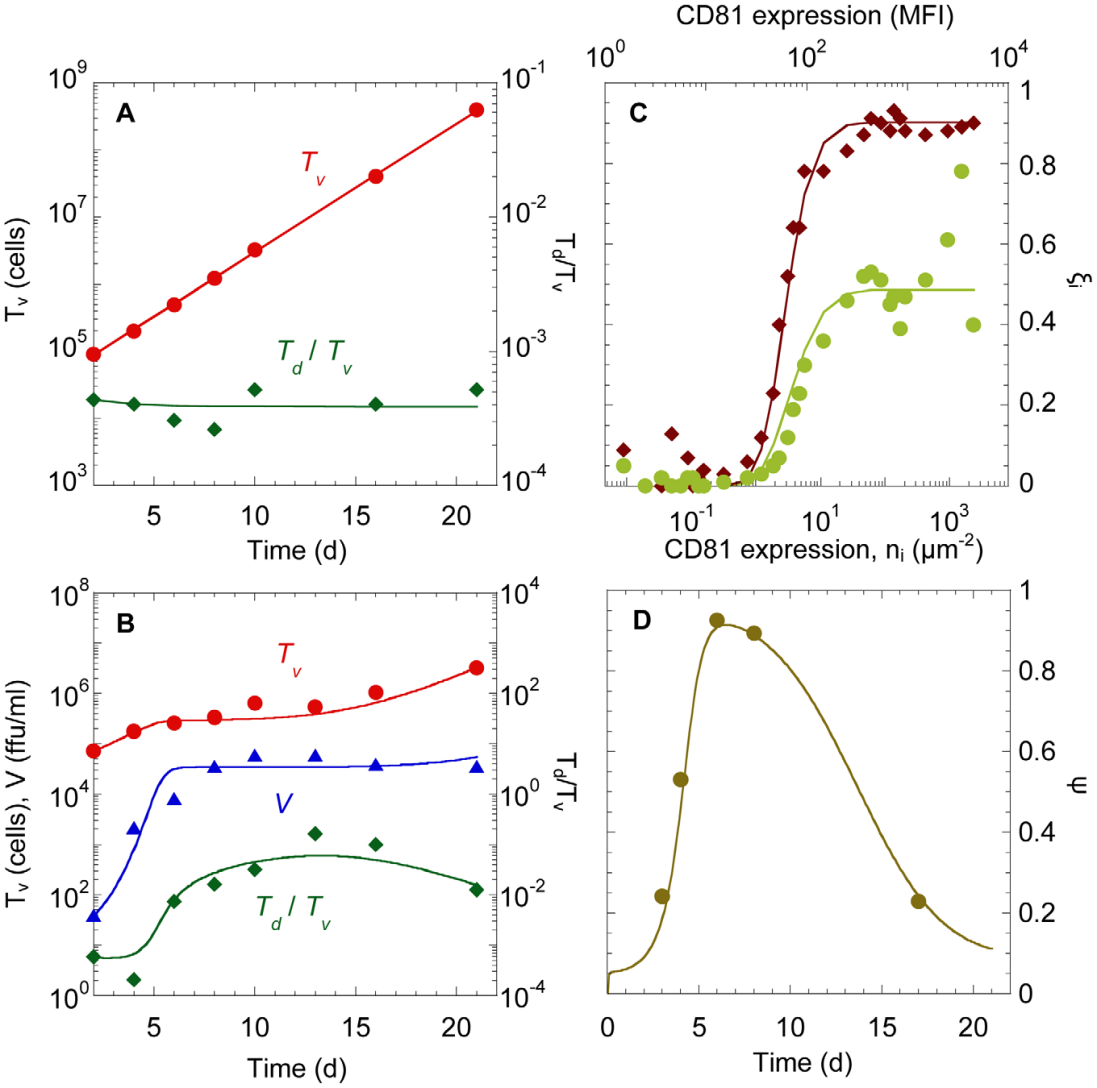
Comparisons with experiments. (A) Fits of model predictions (lines) to data [15] (symbols) of the time-evolution of the population of viable cells, 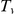, and the ratio of the populations of dead and viable cells, 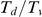, in the absence of infection using 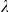 and 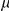 as adjustable parameters. Initial conditions: 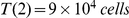 and 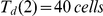. (B) Fits of model predictions (lines) to data (symbols) [15] of the time-evolution of 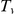, 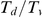, and the viral titre, 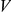, using 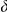, 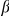, 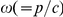, and 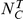 as adjustable parameters and with 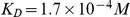. Initial conditions: 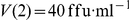; 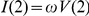; 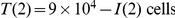; 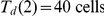. (C) Fits of model predictions (lines) of fractions of cells infected in sub-populations with distinct CD81 expression levels, 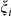, at day 3 post-infection to data [13] (symbols) using 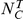, 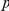 and 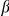 as adjustable parameters and with 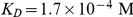. Initial conditions: 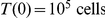; 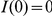; 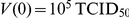 (MOI∼1; circles) or 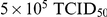 (MOI∼5; diamonds). Fits with other values of 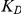 are presented in Fig. S4. The best-fit parameters are in Tables S1 and S2. (D) Model prediction (line) of the time-evolution of the fraction of cells infected, 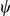, compared with data [14] (symbols). Initial conditions used: 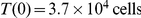; 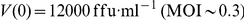; 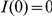. Other parameters are the same as in (A). Comparisons with other values of 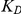 are presented in Fig. S4.

We considered first the experiments of Zhong et al. [15], who measured the kinetics of the growth of Huh-7.5.1 cells with and without exposure to JFH-1 virions. We solved model equations using the initial CD81 distribution corresponding to Huh-7.5 cells [13] (Methods) (Fig. S2). We fit model predictions of the time-evolution of the population of viable cells, 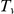, and of the ratio of the populations of dead and viable cells, 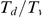, simultaneously to the corresponding experimental data in the absence of infection (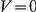). Our model provided good fits to the data and yielded estimates of the proliferation and death rates, 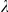 and 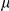, of uninfected Huh-7.5.1 cells (Fig. 3A). It follows from our model that in the absence of infection (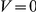), 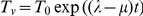 and 
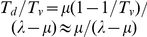
, where 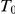 is the population of cells at the start of infection. In agreement, 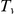 rose linearly on a semi-log plot and the ratio 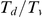 remained nearly constant (Fig. 3A).

Using 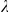 and 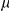 estimated thus and recognizing that viral production and clearance rapidly attain pseudo-steady state (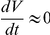, which implies that 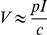), we next fit our predictions of the time-evolution of 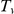, the ratio 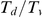, and the viral load, 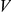, simultaneously to the corresponding measurements during infection (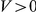) using 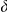, 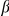, 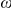 (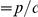), and 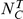 as adjustable parameters for different values of 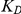. (We employed a truncated Gaussian approximation to the Poisson probability for 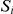 (Fig. S3).) Model predictions again provided good fits to the data (Fig. 3B and Fig. S4A) indicating the ability of our model to capture experimental observations quantitatively.

The data were consistent with the triphasic viral kinetics predicted by our model. 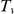 displayed an initial rise followed by a plateau and a subsequent rise (Fig. 3B). 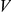 displayed an initial rise and a plateau. The ratio 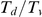 rose initially, attained a maximum, and then declined. In our model calculations, both 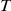 and 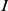 rose in the first phase (Fig. 2), in agreement with the initial rise of 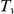, which is the sum of 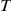 and 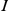, in the experiments. In the second phase, 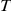 declined primarily due to infection, giving rise to 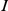, which maintained a nearly constant sum of 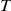 and 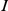, consistent with the plateau in 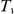. In the third phase, 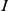 remained nearly constant or rose marginally, whereas 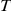 rose significantly, in agreement with the subsequent rise of 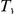. Similarly, the rise of 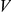 in the second phase followed by the plateau were also in agreement with the trends predicted (Fig. 2). In the first phase, however, 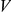 initially declined and then rose as 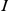 increased (Fig. 2). The data considered here were collected from day 2 post-infection (Fig. 3B), by which period pseudo-steady state between viral production and clearance is expected to be established and the initial decline of 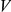 is therefore not expected to be observed. Finally, the initial rise of 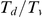 corresponded to the rise of 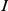 in the first and second phases; 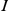 had lifespans shorter than 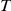 due to virus-induced cytopathicity so that higher values of 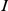 resulted in greater 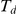. The subsequent drop in 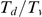 corresponded to the rise of 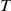 in the third phase, which increased 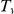 and also lowered the overall rate of cell death.

#### Threshold number of E2-CD81 complexes necessary for entry

**Figure 4:**
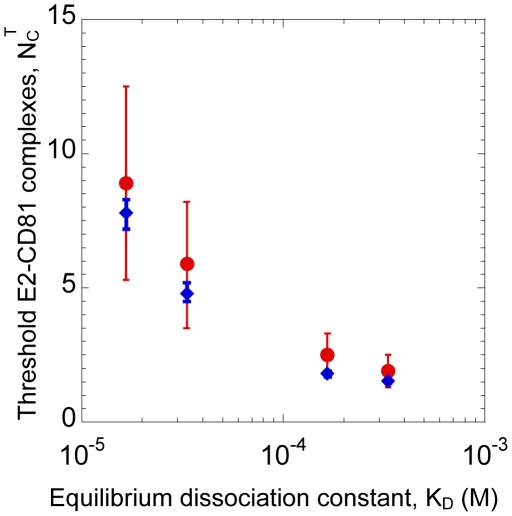
Threshold number of E2-CD81 complexes. Estimates of 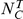 for different values of 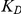 obtained from fits of model predictions to the data of Zhong et al. [15] (circles) and Koutsoudakis et al. [13] (diamonds) (Fig. S4). Error bars are 95% confidence intervals.

From the above fits, for each value of 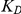 in the range 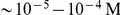, we obtained a corresponding best-fit estimate of 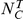 (Fig. 4, Table S1). For example, 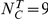 (95% CI: 6–13) when 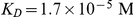 and 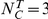 (95% CI: 2–4) when 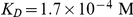. The best-fits thus suggested that depending on the affinity of E2 for CD81, between 1 and 13 E2-CD81 complexes across a virus-cell pair were necessary for the entry of JFH-1 virions into Huh-7.5.1 cells.

#### Robustness of parameter estimates

To test the robustness of our estimate of 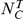, we applied our model to the analysis of two independent datasets of viral entry and kinetics in vitro. Koutsoudakis et al. [13] exposed an equal mixture of Huh7-Lunet and Lunet/CD81 cells to Jc1 virions at two different MOIs and measured the fractions of cells infected in sub-populations with distinct CD81 expression levels at day 3 post-infection. To compare our model predictions with their data, we employed the initial CD81 distribution obtained by averaging the distributions on Huh7-Lunet and Lunet/CD81 cells (Fig. S2) and predicted the fraction of cells infected in cell sub-populations with distinct CD81 expression levels, 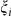. We fixed 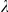, 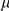, and 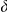 to the values estimated above (Table 1). The two initial MOIs employed would lead to different pseudo-steady states between viral production and clearance; i.e., 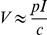 would still hold, but the values of 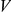 and 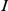 would be different when the pseudo steady state is reached in the two cases. To account explicitly for the different initial MOIs employed, we therefore relaxed the pseudo-steady state approximation in our model. We obtained 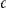, the viral clearance rate, by fitting our model predictions without the pseudo-steady state approximation to the above data of Zhong et al. [15] (Fig. S5). We then fit our predictions of 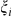 to the data of Koutsoudakis et al. [13] with both the initial MOIs simultaneously using the appropriate initial conditions and 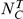, 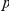 and 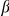 as adjustable parameters for different values of 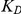. In effect, we assumed that the differences in the viral clones, JFH-1 and Jc1, and the cell lines, Huh7.5 and the combination of Huh7-Lunet and Lunet/CD81, affected the viral production rate and/or the infection rate.

**Table 1:** Summary of model parameters and their values employed.

Parameter	Description	Value (95% CI)^a^	Source
*λ*	Proliferation rate of target cells	0.44 (0.43–0.46) d^−1^	Best-fit (Fig. 3A)
*μ*	Death rate of target cells	1.7 (1.4–2.0)×10^−4^ d^−1^	Best-fit (Fig. 3A)
*δ*	Death rate of infected cells	1.1 (0.4–1.8)×10^−2^ d^−1^	Best-fit (Fig. 3B)
*β*	Infection rate of cells with excess CD81 expression	1.2 (0.6–1.8)×10^−4^ ml•(ffu•d)^−1^	Best-fit (Fig. 3B)
*ω*	Ratio of viral production rate per cell (*p*) and clearance rate (*c*)	0.12 (0.06–0.19) ffu•ml^−1^	Best-fit (Fig. 3B)
*c*	Clearance rate	23.2 d^−1^	Best-fit (Fig. S5)
*K_D_*	Equilibrium dissociation constant of the E2-CD81 binding reaction	10^−4^–10^−5^ M	Varied
*n_E_*	Number density of E2 molecules on HCV	2.3×10^4^ µm^−2^	[53]
*A*	Virus-cell contact area	1.3×10^−3^ µm^2^	[54]

a Typical values employed. Variations are indicated in the text and in figure legends.

Model predictions again provided excellent fits to the data (Fig. 3C and Fig. S4B). For CD81 expression levels 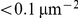, 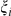 remained negligible for both MOIs, indicating that these subpopulations were refractory to infection. For higher expression levels, 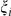 increased with CD81 expression and reached a plateau at an expression level of 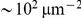, beyond which viral entry was not limited by CD81. The plateau attained different values for the two MOIs, as expected from the different underlying viral kinetics. With MOI of 5, ∼90% of the cells with excess CD81 were infected by day 3, whereas with MOI of 1, ∼50% of the cells with excess CD81 were infected. The resulting estimates of 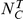 were in close agreement with the estimates obtained above (Fig. 4 and Table S2), giving us confidence in our model and our estimates of 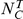. We note that model predictions of the total percentage of cells infected, 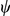, at MOI 5 and 1 (∼31% and ∼23%, respectively) were lower than the corresponding measurements (57% and 29%). We found that altering the initial mixture (1∶4 Huh7-Lunet and Lunet/CD81 cells) or the initial distribution of CD81 expression brought our predictions (∼50% and ∼30%, respectively) close to the experiments. Fits to the data and the resulting estimates of 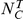 remained unaltered; the only differences were in the estimates of 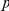 and 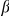, again indicating the robustness of the estimates of 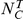.

We considered finally the experiments of Tscherne et al. [14], where the fraction of Huh 7.5 cells infected with J6/JFH virus was measured as a function of time following the onset of infection. We solved our model equations without any adjustable parameters and with the appropriate initial conditions and predicted the time-evolution of the fraction of cells infected, 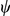 (Fig. 3D). Following the onset of infection, 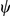 increased and reached a maximum of 90% at day 5 post-infection. Subsequently, cells refractory to infection began to outgrow the susceptible population and 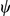 decreased, reaching ∼20% by day 17 post-infection. Our model captured the measured evolution of 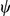 quantitatively (Fig. 3D and Fig. S4C). The agreement between model predictions without any adjustable parameters and experimental observations represented a successful validation of our model as well as the parameter estimates obtained above.

## Discussion

Analysis of experimental data using mathematical models has provided crucial insights into disease pathogenesis and the effectiveness of drugs and established guidelines for rational optimization of therapy for HCV infection [21]–[31], [33], [34]. The recent development of cell culture systems that allow persistent HCV infection in vitro [35]–[38] has yielded a wealth of new data on HCV replication, evolution, and the impact of drugs. Analysis of this data is expected to provide further insights into HCV pathogenesis and outcomes of therapy, but has been precluded by the lack of mathematical models of HCV viral kinetics in vitro. Indeed, significant efforts are underway to construct models of the intracellular replication and evolution of HCV with the aim of elucidating the activity of direct acting antiviral drugs [39]–[41]. Here, we have constructed a mathematical model of HCV viral kinetics in vitro. Applying it to the analysis of data from several recent cell culture studies, we obtained quantitative insights into the molecular requirements of HCV entry. We estimated that depending on the binding affinity of E2 and CD81, between 1 and 13 E2-CD81 complexes are necessary for HCVcc entry into human hepatoma-derived cells.

Our estimate provides a quantitative guideline for the optimal usage of drugs and vaccines that target the E2-CD81 interaction: A potent drug or vaccine must ensure that not more than 1–13 E2-CD81 complexes are formed across a virus-cell pair in order to prevent viral entry. This guideline assumes significance as drugs that target the E2-CD81 interaction are under development [11], [12] and may become part of future treatments involving direct acting antiviral agents that seek to overcome the limitations of the current interferon-ribavirin-based treatments [42]. Further, a recent analysis of the HCV quasispecies in six patients who underwent liver transplantation revealed that viral strains capable of more efficient entry, achieved through modulation of the CD81 dependence of viral entry, were selected following liver transplantation [8]. Blocking E2-CD81 interactions effectively, for which our estimate presents a quantitative criterion, may thus be a promising avenue to prevent graft reinfection following liver transplantation.

In an earlier study, Koutsoudakis et al. [13] estimated that a cell must express >70000 CD81 molecules to allow HCV entry. This threshold was identified as follows. In independent experiments, cells with widely varying distributions of CD81 were exposed to HCV at low MOI in culture and the percentage of cells infected at day 5 was measured. This latter percentage was found to correlate well with the percentage of cells initially expressing >70000 CD81 molecules/cell. For instance, with Huh7.5 cells, the percentage of cells infected at day 5 was 91.5 and the percentage expressing >70000 CD81 molecules/cell at day 0 was 93, and with Huh7-Lunet cells, which express fewer CD81 molecules than Huh7.5 cells, the percentages were 11.2 and 17, respectively. Based on this correlation, Koutsoudakis et al. suggested 70000 CD81 molecules/cell as the threshold for entry. Our present analysis suggests that the underlying viral kinetics may render this estimate an upper bound. Virus-induced cytopathicity in culture would result in the loss of susceptible cells and therefore a continuous decrease in the frequency of susceptible cells with time. Consequently, the percentage of cells susceptible initially is expected to be higher than the percentage of cells susceptible–and hence even higher than the percentage infected–at day 5 post-infection. The higher percentage of cells susceptible initially would therefore imply a threshold smaller than 70000 molecules/cell. This is also evident in the experiments of Koutsoudakis et al., where cells with lower CD81 expression (MFI∼50) than 70000 molecules/cell (MFI∼100) were infected (see Fig. 3C). From our analysis, we identified the threshold number of E2-CD81 complexes and not CD81 molecules necessary for entry. Because stochastic events can result in the formation of the requisite number of complexes, cells expressing few CD81 molecules have small but nonzero susceptibilities to infection. Indeed, we found using parameters employed in Fig. 3D that a small percentage of cells expressing as few as ∼10000 CD81 molecules was infected by day 5. More recently, Zhang et al. [18] have argued that a substantially smaller expression level than suggested by Koutsoudakis et al. may suffice for entry and that CD81 may also be necessary, perhaps at higher expression levels, for post-entry events. Our model does not distinguish between entry and post-entry requirements of CD81. Our estimate of 1–13 E2-CD81 complexes defines successful infection of a cell and combines the requirements for entry and any post-entry steps [17], [18].

Our model yielded good fits to data with 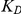 in the range 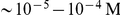, which is higher than the value, 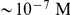, determined using recombinant E2 and soluble CD81 [43]. (Fits with 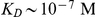 were poor (not shown).) This discrepancy may be because the binding affinity when the proteins are in solution may be different from that when the proteins are restricted to membranes [44], recombinant E2 may not accurately mimic the true E2-CD81 interaction [45], and/or only a fraction of the CD81 may lie outside tetraspanin-enriched microdomains and/or be associated with CLDN1 and therefore available for binding E2 [10], [46]. The E2-CD81 binding affinity in situ remains to be determined. Our model yielded best-fit values of the threshold number of complexes, 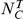, that decreased as 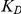 increased (Fig. 4). For a given CD81 expression level, the mean number of E2-CD81 complexes formed decreased as 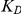 increased, lowering susceptibility (Eqs. (4) and (5)). Decreasing 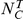 restored this susceptibility (Fig. 2B inset and Fig. S6), thus ensuring that the resulting viral kinetics was conserved and in agreement with data. This does not imply, however, that viral strains with lower E2-CD81 affinity (higher 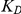) would require fewer E2-CD81 complexes for entry. On the contrary, given a value of 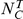, our model predicts that a cell would be less susceptible to entry of viral strains with higher 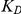.

Our model was designed to mimic experiments that examined the influence of CD81 expression on viral entry and kinetics [13]–[15]. In these experiments, cells with high CD81 expression were preferentially infected and lost due to virus-induced cytopathicity, and cells with low CD81 expression, refractory to infection, eventually dominated the culture, suggesting that CD81 expression limited entry. Accordingly, our model assumed that other entry receptors were not limiting. Our model then predicted triphasic viral kinetics in vitro, in agreement with experiments. We note that the origins of the triphasic pattern here are distinct from the triphasic viral load decline in some patients undergoing combination therapy, the latter due to liver homeostatic mechanisms [25]. Further, the triphasic kinetics is a short-term phenomenon (∼2–3 weeks). Over longer periods, viral evolution may alter the kinetics substantially [15], which our model does not consider. Nonetheless, our model can be readily adapted to the scenario where a receptor other than CD81 is limiting and may thus serve to quantify the requirements of that receptor for HCV entry.

We recognize a few additional simplifications in our model. First, our model ignored cell-cell transmission of infection. CD81 appears to be necessary for direct cell-cell transmission [47]. If the susceptibility of a cell to cell-cell transmission depends on CD81 expression in a manner similar to its susceptibility to viral entry, which remains to be ascertained, then we can show that our model with the pseudo-steady state approximation, 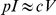, implicitly accounts for cell-cell transmission: the net infection rate from both modes, 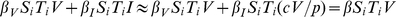, is in agreement with our model (Eq. (1)) with 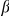 an effective infection rate constant that lumps the rate constants of infection by free virions, 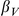, and cell-cell transmission, 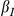. Second, our model assumes that reaction equilibrium is attained rapidly compared to viral entry and that the diffusion of CD81 on the target cells continually replenishes the free CD81 in the virus-cell contact region lost due to binding. Accordingly, our model predicts an upper bound on the mean number of E2-CD81 complexes formed in the contact region. Thus, if CD81 diffusion or its binding with E2 were rate limiting, a threshold smaller than 1–13 E2-CD81 complexes is expected to describe the data we considered. Finally, we ignored the splitting of cell culture at confluence. We employed the data at day 3 post-infection from Koutsoudakis et al. [13], when cells are not expected to have reached confluence. Further, the best-fit parameter estimates obtained from the data of Koutsoudakis et al. [13] were close to those from Zhong et al. [15] (Fig. 4). Also, accounting for splitting did not significantly alter our comparisons with the data of Tscherne et al. [14] so long as the splitting was performed after day 5 post-infection (Fig. S7). Also, including a logistic term to limit the proliferation of cells as they approached confluence did not alter our parameter estimates significantly (not shown). Nonetheless, that model predictions described several independent experimental observations quantitatively indicated that even with the above simplifications our model captured the essential features of HCV viral kinetics in vitro successfully. At the same time, the simplifications restricted model parameters to a number that allowed robust parameter estimation through fits to available data.

## Methods

### Model of HCV kinetics in vitro

We considered *in vitro* experiments where a population of uninfected cells, 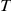, is exposed to a population of HCVcc virions, 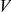. We divided the cells into 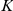 subpopulations, denoted 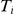, where 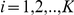, with cells in each subpopulation expressing CD81 in a range 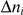 around 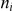 molecules per unit area. At the start of infection (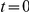), the variation of 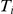 with 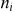 was determined from a known distribution, 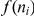, of CD81 expression levels across cells (Fig. 1). The following equations described the ensuing viral kinetics (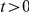):
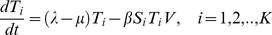
(1)

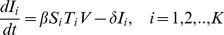
(2)

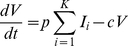
(3)Here, 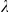 and 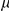 are the proliferation and death rates of 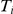. 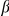 is the infection rate of cells expressing excess CD81. 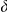 is the death rate of 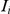. Following observations of HCV-induced cell cycle arrest in vitro [32], [48], we neglected the proliferation of 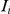. 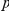 and 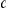 are the per cell production rate and the clearance rate of free virions, respectively. Here, 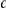 represents the combined rate of the natural degradation of virions, the loss of viral infectivity, and the loss of virions due to entry and attachment [49], [50]. For simplicity, we assumed 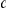 to be a constant.

To determine 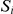, we considered a cell 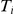, with CD81 expression level 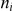, closely apposed to a virion with 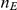 E2 molecules per unit area. We assumed, as with HIV [20], that the E2-CD81 interactions across the virus-cell interface attain equilibrium well before viral entry. If 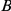 is the surface density of E2-CD81 complexes, 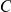 that of unbound CD81 and 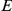 that of unbound E2 molecules in the contact area, then at equilibrium 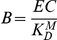, where 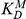 is the equilibrium dissociation constant of E2-CD81 complexes when the proteins are restricted to membranes. From Bell's analysis, 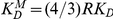, where 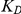 is the equilibrium dissociation constant when the proteins are in solution and 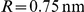 is the encounter distance between the proteins for bond formation [51]. The virus-cell contact area, 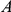, is small compared to the surface area of the cell. Further, free CD81 can diffuse on the cell membrane and therefore be recruited to the contact area. Consequently, 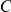 is expected not to decrease substantially below 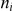, as suggested also by an independent reaction-diffusion model [52]. In contrast, the viral surface area is comparable to 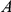 and assuming E2 to be less mobile than CD81, it follows that the surface density of E2 in the contact area obeys the species balance equation: 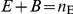. Under the latter two constraints, the mean number of complexes formed across the virus-cell contact at equilibrium, 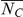, is given by
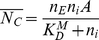
(4)


We recognized next that the E2 expression level on the virion and the virus-cell contact area are subject to stochastic variations. We assumed therefore that the number of complexes formed during a virus-cell contact, 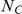, follows a Poisson distribution with mean 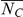. Viral entry (and subsequently infection) occurred if 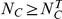, where 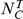 is the threshold number of E2-CD81 complexes necessary for HCV entry. The probability that 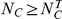,
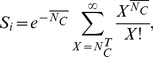
(5)thus yielded the relative susceptibility to infection of a cell with CD81 expression level 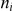. (The Poisson distribution does allow 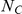 to exceed the limit of 180 set by the number of E2 molecules present on a virion, but in all our calculations 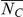 remained fewer than 30 (Fig. S8) so that the probability that 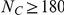 was negligibly small.)

Equations (1)–(5) yielded a model of HCV kinetics in vitro that accounted explicitly for the dependence of viral entry on the CD81 expression level on cells.

### Model parameters and calculations

We solved model equations using a computer program written in MATLAB and computed quantities measured experimentally, namely, the time-evolution of the populations of uninfected and infected cells, 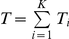 and 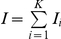, the viral titre, 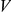, the fraction of cells infected, 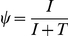, the fraction of cells infected within each subpopulation, 
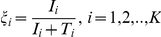
, and the populations of viable and dead cells, 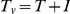 and 
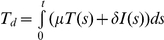
, respectively.

We employed the following parameter values and initial conditions: 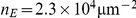 corresponding to 180 E2 molecules on a virion with average diameter 50 nm [53]; 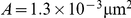, corresponding to a virus-cell contact radius of 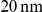 [54]; the target cell diameter was 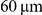 [17]; 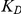 was varied over the range 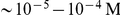 (see above). The initial CD81 expression was assumed to follow the log-normal distribution, 
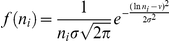
, where 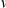 and 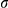 were the mean and standard deviation of 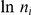. For comparisons with experimental data, the initial distributions were obtained from measurements (Fig. S2; see below). Thus, the initial cell subpopulations, 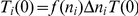, where 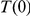 was the total initial target cell population. We divided the range of CD81 expression levels into 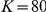 intervals, which determined 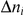; finer discretisation did not improve the accuracy of our solution (Fig. S1). The remaining parameters, estimated from fits to data, are listed in Tables S1 and S2. We have summarized model parameters in Table 1.

### Data and fitting

We considered data from three recently published cell culture studies of HCV kinetics. First, we considered data of Zhong et al. [15], where Huh-7.5.1 cells were exposed to JFH-1 virions and the kinetics of infection followed for 21 d. Specifically, we employed the data of the time-evolution of the supernatant infectivity, the population of attached (viable) cells, and the ratio of the populations of floating (dead) and attached cells, the latter two datasets with mock infection as well as with HCVcc infection (Fig. S1 in [15]). Second, we considered data of Koutsoudakis et al. [13], where a 1∶1 mixture of Huh7-Lunet and Lunet/CD81 cells was exposed to HCVcc Venus-Jc1 virus and the fractions of cells infected in subpopulations with distinct CD81 expression levels were measured after 72 h. The initial viral population was at an MOI of ∼5 and ∼1 TCID_50_/cell (where TCID_50_ is the 50% tissue culture infective dose), respectively, in two independent experiments (Fig. 5 in [13]). Third, we considered the data of Tscherne et al. [14], where Huh7.5 cells were exposed to J6/JFH virus and the time-evolution of the fraction of cells infected as well as of the distribution of CD81 expression levels across cells was followed for 17 d (Fig. 8 in [14]).

The CD81 expression on cells is usually measured in terms of fluorescent intensity. To convert the measurements to CD81 surface densities, we adopted the following procedure. Measured distributions of the CD81 expression level on Huh-7.5 (silRR) cells were digitized from Zhang et al. [55] and on Huh-7.5 cells from Koutsoudakis et al. [13]. The log-normal distribution, 
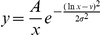
, yielded good fits to the data (Fig. S9). The best-fit parameter values (95% CI) were 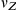 = 4.29 (4.27–4.31) and 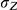 = 0.45 (0.43–0.47) for the data of Zhang et al. and 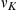 = 6.1 (6.07–6.12) and 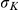 = 0.7 (0.67–0.72) for the data of Koutsoudakis et al. Because the same cell lines were used, the underlying distributions of CD81 expression are expected to be similar in the two experiments. The differences in the reported fluorescence intensities may therefore be attributed to different conversions of CD81 surface density to fluorescence intensity in the two measurements. To unravel the underlying distribution, we assumed that log fluorescence intensity was linearly proportional to log CD81 surface density (or that the fluorescence intensity had a power law dependence on the surface density). Thus, a cell with CD81 surface density 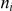 would yield a fluorescence intensity 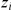 and 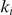 in the measurements of Zhang et al. and Koutsoudakis et al., respectively, such that 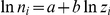 and 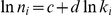, where *a*, *b*, *c*, and *d* are constants. To determine the latter constants, we employed the following observations: The mean surface density of CD81 for the Huh-7 cell line is 2×10^5^ molecules/cell [3], which corresponded to a fluorescence intensity of 48 units in the measurements of Zhang et al., so that 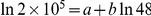. Also, Koutsoudakis et al. measured the number of CD81 molecules/cell corresponding to a fluorescence intensity of 100 units and found it to be 7×10^4^ molecules/cell, so that 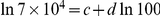. Further the above relationship between fluorescence intensity and CD81 expression also implied that 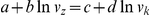 and 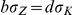. Solving the latter equations using the best-fit parameters above, we obtained *a* = 2.8, *b* = 2.43, *c* = 3.96 and *d* = 1.56, which enabled conversion of measured fluorescence intensities to CD81 surface densities, 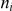 (Fig. S2). The measured distributions in terms of counts versus fluorescence intensities were then converted to probability distributions, 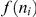 versus 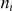, by normalizing the counts such that the areas under the 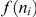 versus 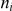 curves equalled unity. The resulting distributions (Fig. S2) were employed as the initial CD81 distributions for our fits to data (Fig. 3).

We digitized data using Engauge digitizer and fit model predictions to data using the nonlinear regression tool NLINFIT in MATLAB.

## Supporting Information

Figure S1
**Sensitivity to model parameters.** Time-evolution of (A) uninfected cells, 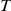, (B) infected cells, 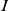, (C) viral load, 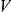, and (D) the fraction of cells infected, 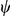, obtained by varying (I) the equilibrium dissociation constant, 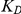 = 1.7×10^−5^ M (thick solid line), 3.3×10^−5^ M (thin solid line), and 1.7×10^−4^ M (dashed line), (II) the mean of the log initial CD81 expression level, 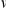 = 29.8 (thick solid line), 31.8 (thin solid line), and 33.8 (dashed line), (III) the initial viral load, 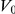 = 40 ffu·ml^−1^ (thick solid line), 400 ffu·ml^−1^ (thin solid line), and 4000 ffu·ml^−1^ (dashed line), (IV) the infection rate constant, 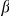 = 1.2×10^−3^ ml·(ffu·d)^−1^ (thick solid line), 1.2×10^−4^ ml·(ffu·d)^−1^ (thin solid line), and 1.2×10^−5^ ml·(ffu·d)^−1^ (dashed line), (V) the viral production rate, 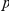 = 0.278 ffu·(ml·d)^−1^ (thick solid line), 2.78 ffu·(ml·d)^−1^ (thin solid line), and 27.8 ffu·(ml·d)^−1^ (dashed line), and (VI) the number of cell sub-populations, 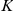 = 40 (thick solid line), 80 (thin solid line), and 120 (dashed line). (The three curves in (VI) are indistinguishable.)(TIF)

Figure S2 **Initial distribution of the CD81 expression level on target cells.** Distribution of the CD81 expression level on (A) Huh-7.5 cells and (B) Huh7-Lunet cells (diamonds) and Lunet/CD81 cells (circles) obtained by digitizing data from Koutsoudakis et al. (2007) J Virol 81:588–598 and converting fluorescence intensities to CD81 surface densities (Methods).
(TIF)

Figure S3
**Approximating the Poisson distribution with a truncated Gaussian distribution.** Model predictions of the susceptibility of cells using the Poisson distribution, 
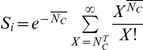
 (dashed line), and an equivalent truncated Gaussian distribution with mean 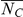 and standard deviation 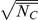, which yields 
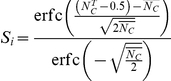
 (solid line), where 
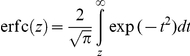
 is the complementary error function, for different values of 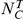 and for (A) 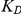 = 1.7×10^−5^ M and (B) 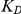 = 3.3×10^−4^ M.(TIF)

Figure S4
**Comparisons of model predictions with data using different values of** ***K_D_*** **.** (A) The fit in Fig. 3B repeated with 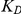 = 1.7×10^−5^ M (thick solid line), 3.3×10^−5^ M (thin solid line), 1.7×10^−4^ M (dashed line), and 3.3×10^−4^ M (dotted line). The resulting best-fit parameter estimates are in Table S1. (The fits for different 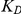 nearly overlap and are indistinguishable.) (B) Fits in Fig. 3C repeated with the above values of 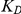. The resulting best-fit parameter estimates are in Table S2. (C) The comparison in Fig. 3D repeated with the above values of 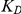 and other parameter values in Table S1.(TIF)

Figure S5
**Fits without the pseudo-steady state approximation.** The pseudo-steady state approximation is relaxed and our model predictions (Eqs. (1)–(5)) are fit to the data in Fig. 3B using the parameter estimates in Table S1 and with viral clearance rate *c* as an adjustable parameter. Initial conditions used are: 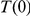 = 3.7×10^4^ cells; 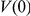 = 400 ffu·ml^−1^; 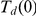 = 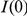 = 0. The resulting estimates of *c* are 21.4, 22.7, 23.2 and 23.8 d^−1^ for 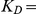
1.7×10^−5^, 3.3×10^−5^, 1.7×10^−4^, and 3.3×10^−4^ M, respectively. (The fits for different 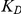 nearly overlap and are indistinguishable.)(TIF)

Figure S6
**Susceptibility of cells predicted using different best-fit parameter combinations.** Dependence of 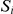 on CD81 expression predicted using the parameter combinations in (A) Table S1 and (B) Table S2 where 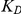 = 1.7×10^−5^ M (thick solid line), 3.3×10^−5^ M (thin solid line), 1.7×10^−4^ M (dashed line), and 3.3×10^−4^ M (dotted line).(TIF)

Figure S7 **Influence of splitting of cell culture at confluence.** Model predictions of the fraction of cells infected without splitting (thick line), splitting at day 6 and day 12 (thin line), and splitting at day 8 and day 12 (dashed line) after the onset of infection compared with the data in Fig. 3D (symbols). Parameters used are the same as in Fig. 3D.
(TIF)

Figure S8
**Mean number and probability of formation of E2-CD81 complexes.** (A) Model predictions of the mean number of E2-CD81 complexes formed, 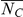, as a function of CD81 expression for 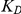 = 1.7×10^−5^ M (solid line), 3.3×10^−5^ M (dashed line), 1.7×10^−4^ M (dashed-dotted line), and 3.3×10^−4^ M (dotted line). (B) Model predictions of the Poisson probability of forming 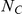 E2-CD81 complexes (Eq. (5)) when 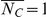 (solid line) and 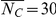 (dashed line).(TIF)

Figure S9
**Conversion of fluorescence intensity to CD81 expression.** Measured distributions of the CD81 expression level on Huh-7.5 (silRR) cells digitized from Zhang et al. (2004) J Virol 78:1448–1445 (diamonds) and Huh-7.5 cells from Koutsoudakis et al. (2007) J Virol 81:588–598 (triangles). Lines are best-fits of the log-normal distribution, 
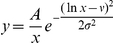
, to the data. The best-fit parameter values (95% CI) are 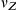 = 4.29 (4.27–4.31) and 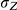 = 0.45 (0.43–0.47) for the data of Zhang et al. and 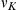 = 6.1 (6.07–6.12) and 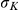 = 0.7 (0.67–0.72) for the data of Koutsoudakis et al.(TIF)

Table S1 **Estimates of model parameters obtained from fits of model predictions to the data in** **Figs. 3B** **and S4A.** 95% confidence intervals are indicated in brackets.
(DOC)

Table S2 **Estimates of model parameters obtained from fits of model predictions to the data in** **Figs. 3C** **and S4B.** 95% confidence intervals are indicated in brackets.
(DOC)
