# Supplementary figures and images for: Mathematical Model of Viral Kinetics In Vitro Estimates the Number of E2-CD81 Complexes Necessary for Hepatitis C Virus Entry
Source: PLoS Comput Biol. 2011 Dec 8;7(12):e1002307. doi: 10.1371/journal.pcbi.1002307 (PMC3234214; doi:10.1371/journal.pcbi.1002307)

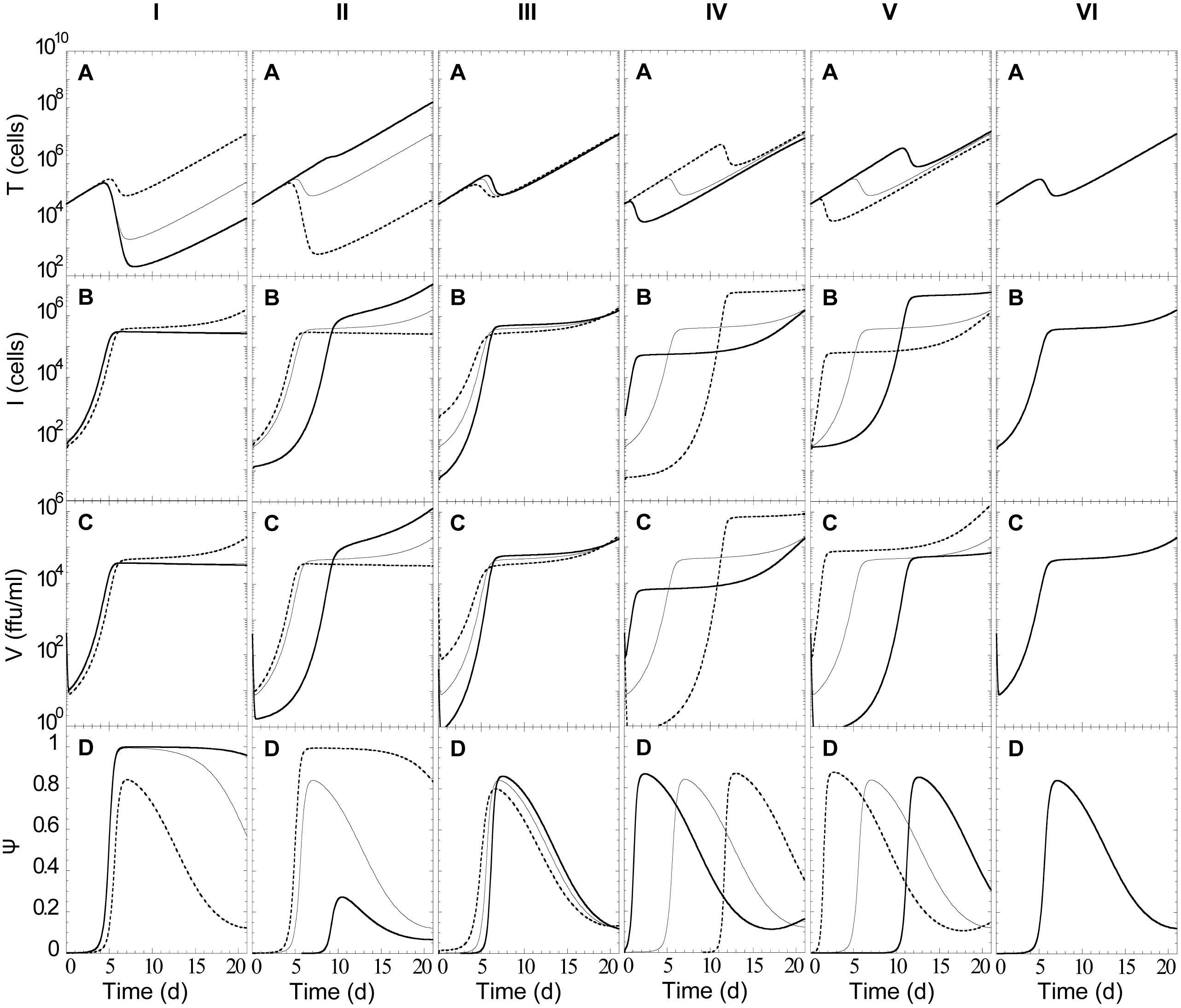

Supplement: Figure S1 — Sensitivity to model parameters. Time-evolution of (A) uninfected cells, , (B) infected cells, , (C) viral load, , and (D) the fraction of cells infected, , obtained by varying (I) the equilibrium dissociation constant, = 1.7×10^−5^ M (thick solid line), 3.3×10^−5^ M (thin solid line), and 1.7×10^−4^ M (dashed line), (II) the mean of the log initial CD81 expression level, = 29.8 (thick solid line), 31.8 (thin solid line), and 33.8 (dashed line), (III) the initial viral load, = 40 ffu·ml−1 (thick solid line), 400 ffu·ml−1 (thin solid line), and 4000 ffu·ml−1 (dashed line), (IV) the infection rate constant, = 1.2×10−3 ml·(ffu·d)−1 (thick solid line), 1.2×10−4 ml·(ffu·d)−1 (thin solid line), and 1.2×10−5 ml·(ffu·d)−1 (dashed line), (V) the viral production rate, = 0.278 ffu·(ml·d)−1 (thick solid line), 2.78 ffu·(ml·d)−1 (thin solid line), and 27.8 ffu·(ml·d)−1 (dashed line), and (VI) the number of cell sub-populations, = 40 (thick solid line), 80 (thin solid line), and 120 (dashed line). (The three curves in (VI) are indistinguishable.) (TIF) [file pcbi.1002307.s001.tif]

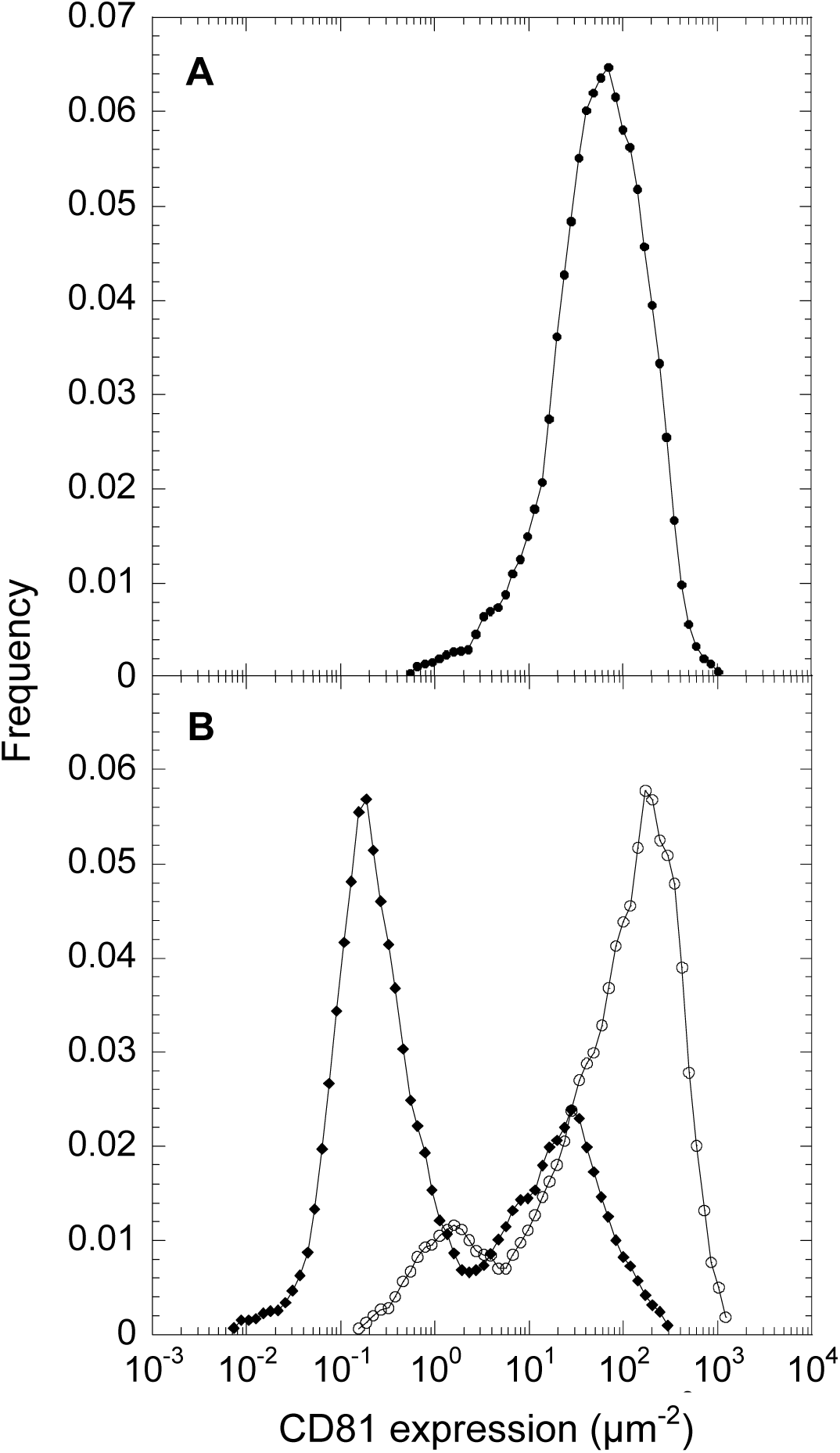

Supplement: Figure S2 — Initial distribution of the CD81 expression level on target cells. Distribution of the CD81 expression level on (A) Huh-7.5 cells and (B) Huh7-Lunet cells (diamonds) and Lunet/CD81 cells (circles) obtained by digitizing data from Koutsoudakis et al. (2007) J Virol 81:588–598 and converting fluorescence intensities to CD81 surface densities (Methods). (TIF) [file pcbi.1002307.s002.tif]

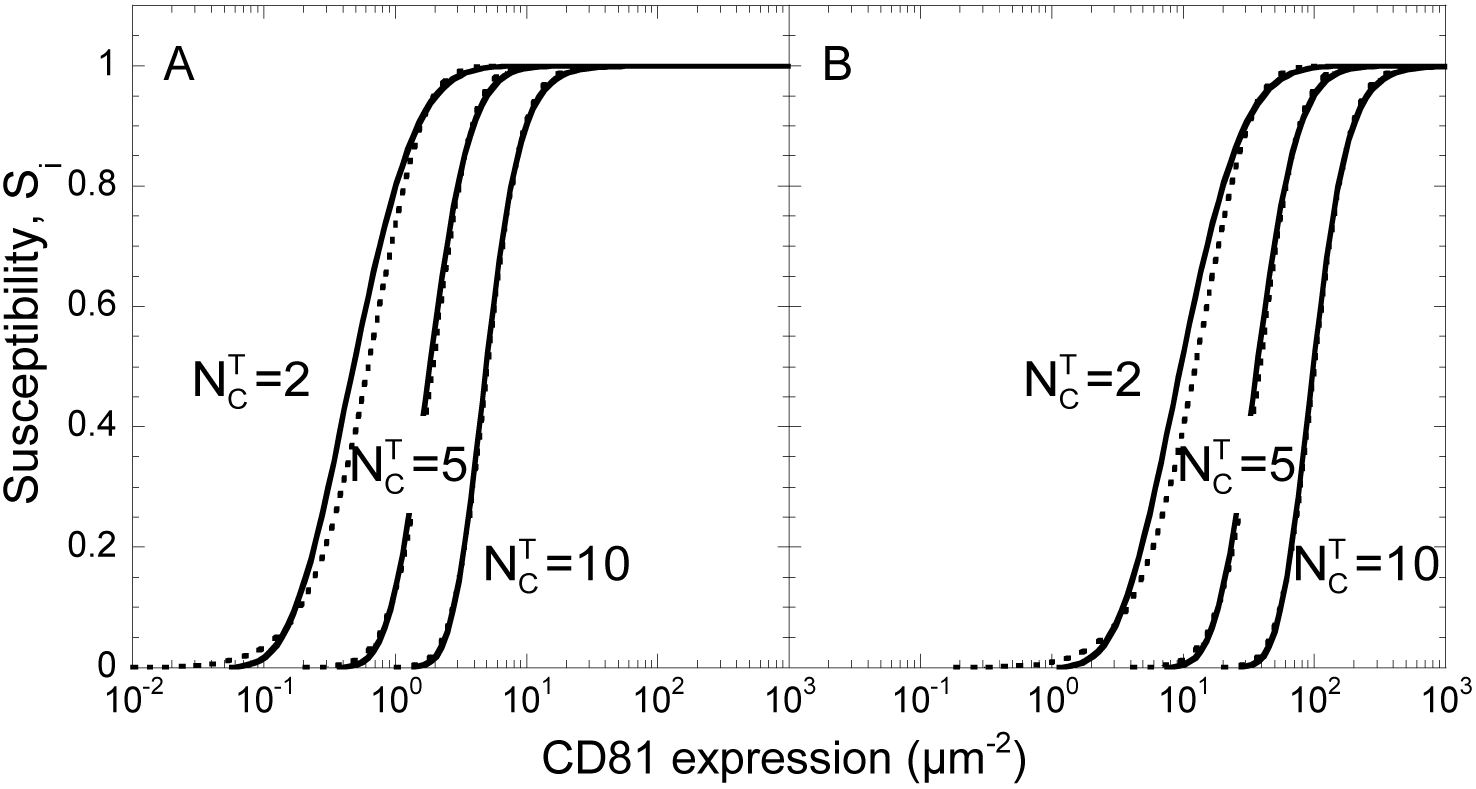

Supplement: Figure S3 — Approximating the Poisson distribution with a truncated Gaussian distribution. Model predictions of the susceptibility of cells using the Poisson distribution, (dashed line), and an equivalent truncated Gaussian distribution with mean and standard deviation , which yields (solid line), where is the complementary error function, for different values of and for (A) = 1.7×10^−5^ M and (B) = 3.3×10^−4^ M. (TIF) [file pcbi.1002307.s003.tif]

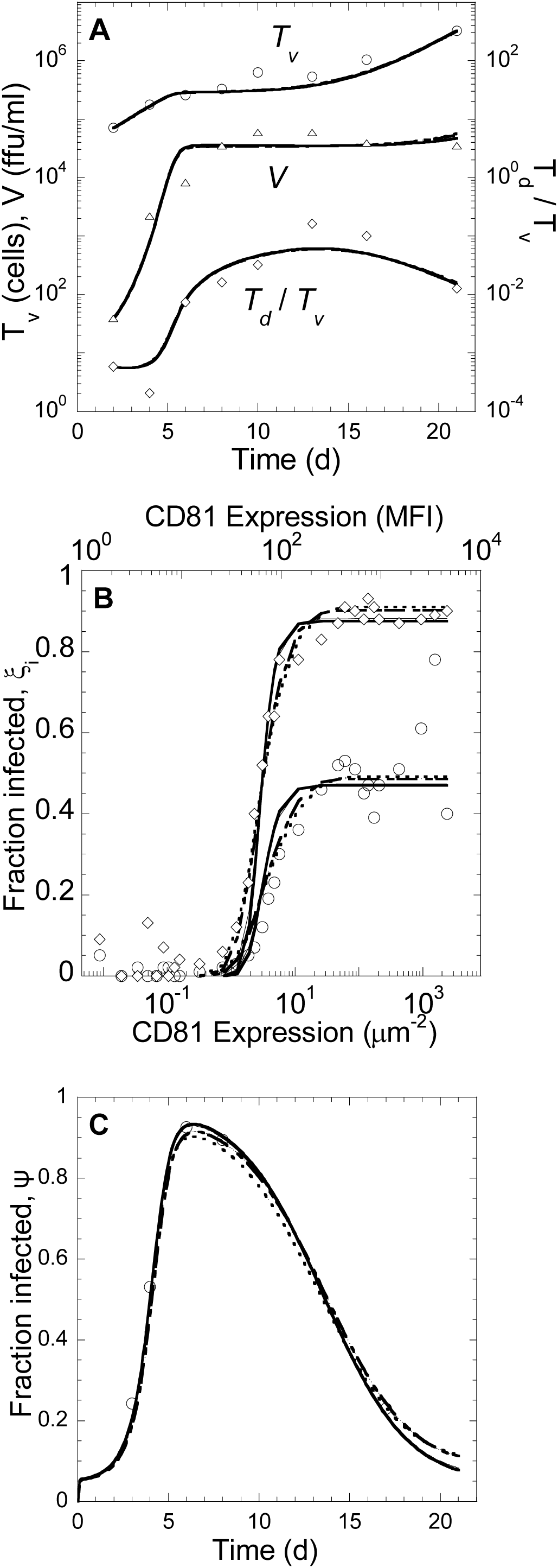

Supplement: Figure S4 — Comparisons of model predictions with data using different values of KD . (A) The fit in Fig. 3B repeated with = 1.7×10^−5^ M (thick solid line), 3.3×10^−5^ M (thin solid line), 1.7×10^−4^ M (dashed line), and 3.3×10^−4^ M (dotted line). The resulting best-fit parameter estimates are in Table S1. (The fits for different nearly overlap and are indistinguishable.) (B) Fits in Fig. 3C repeated with the above values of . The resulting best-fit parameter estimates are in Table S2. (C) The comparison in Fig. 3D repeated with the above values of and other parameter values in Table S1. (TIF) [file pcbi.1002307.s004.tif]

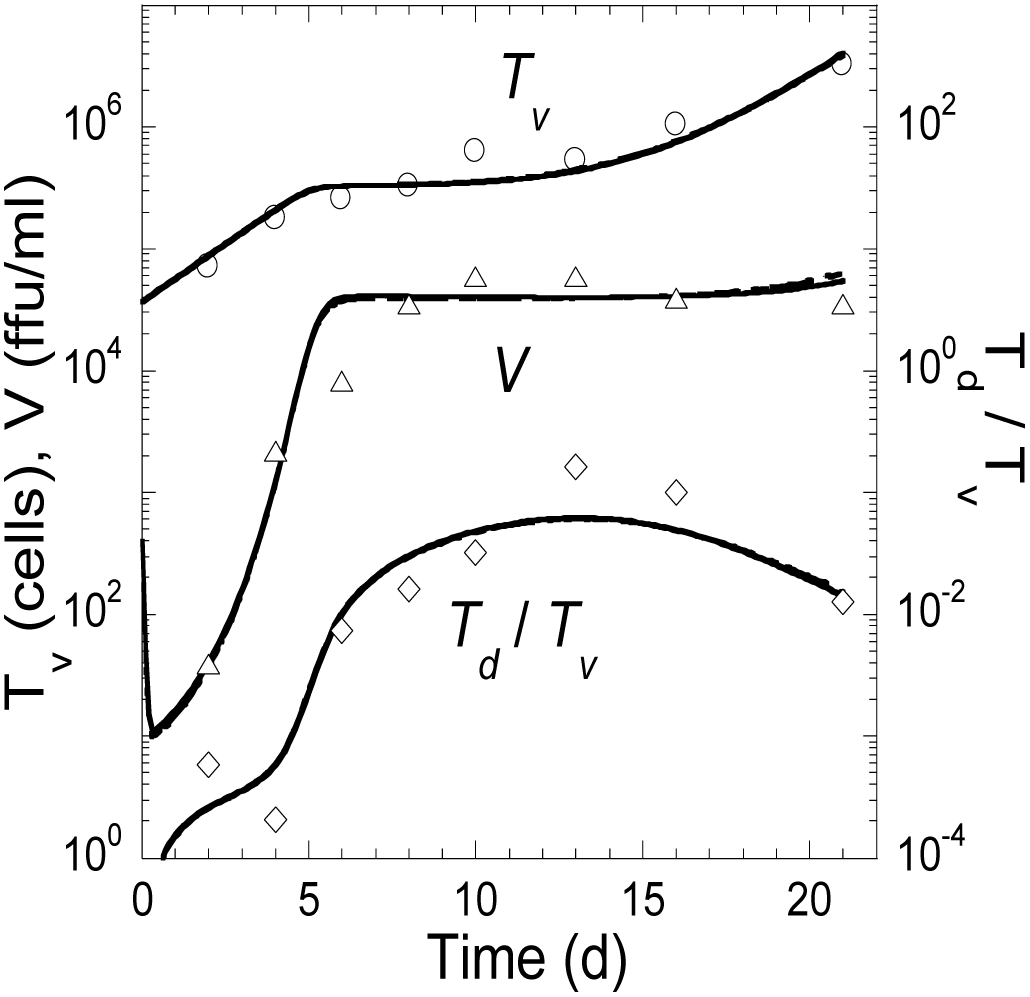

Supplement: Figure S5 — Fits without the pseudo-steady state approximation. The pseudo-steady state approximation is relaxed and our model predictions (Eqs. (1)–(5)) are fit to the data in Fig. 3B using the parameter estimates in Table S1 and with viral clearance rate c as an adjustable parameter. Initial conditions used are: = 3.7×10^4^ cells; = 400 ffu·ml−1; = = 0. The resulting estimates of c are 21.4, 22.7, 23.2 and 23.8 d−1 for 1.7×10^−5^, 3.3×10^−5^, 1.7×10^−4^, and 3.3×10^−4^ M, respectively. (The fits for different nearly overlap and are indistinguishable.) (TIF) [file pcbi.1002307.s005.tif]

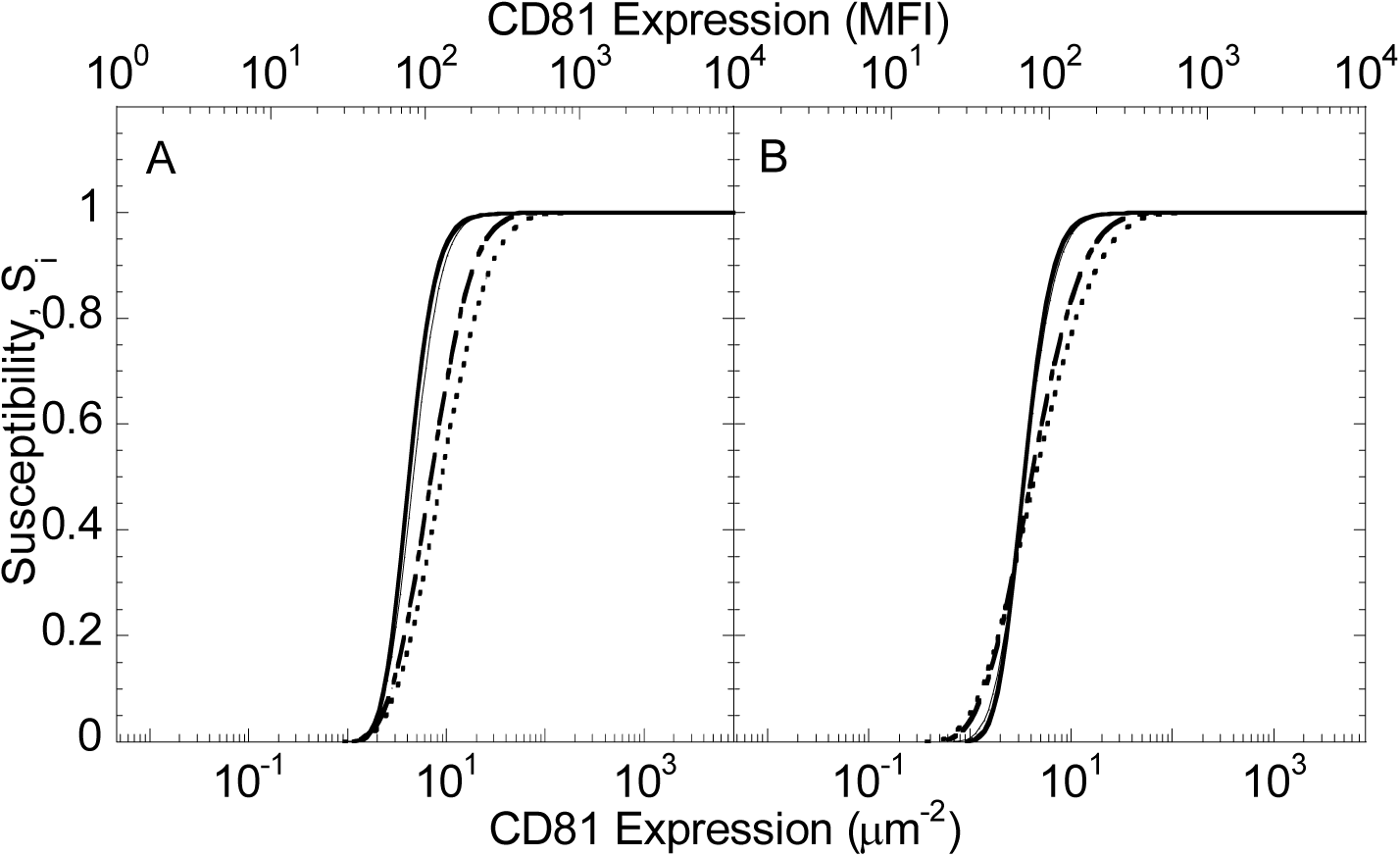

Supplement: Figure S6 — Susceptibility of cells predicted using different best-fit parameter combinations. Dependence of on CD81 expression predicted using the parameter combinations in (A) Table S1 and (B) Table S2 where = 1.7×10^−5^ M (thick solid line), 3.3×10^−5^ M (thin solid line), 1.7×10^−4^ M (dashed line), and 3.3×10^−4^ M (dotted line). (TIF) [file pcbi.1002307.s006.tif]

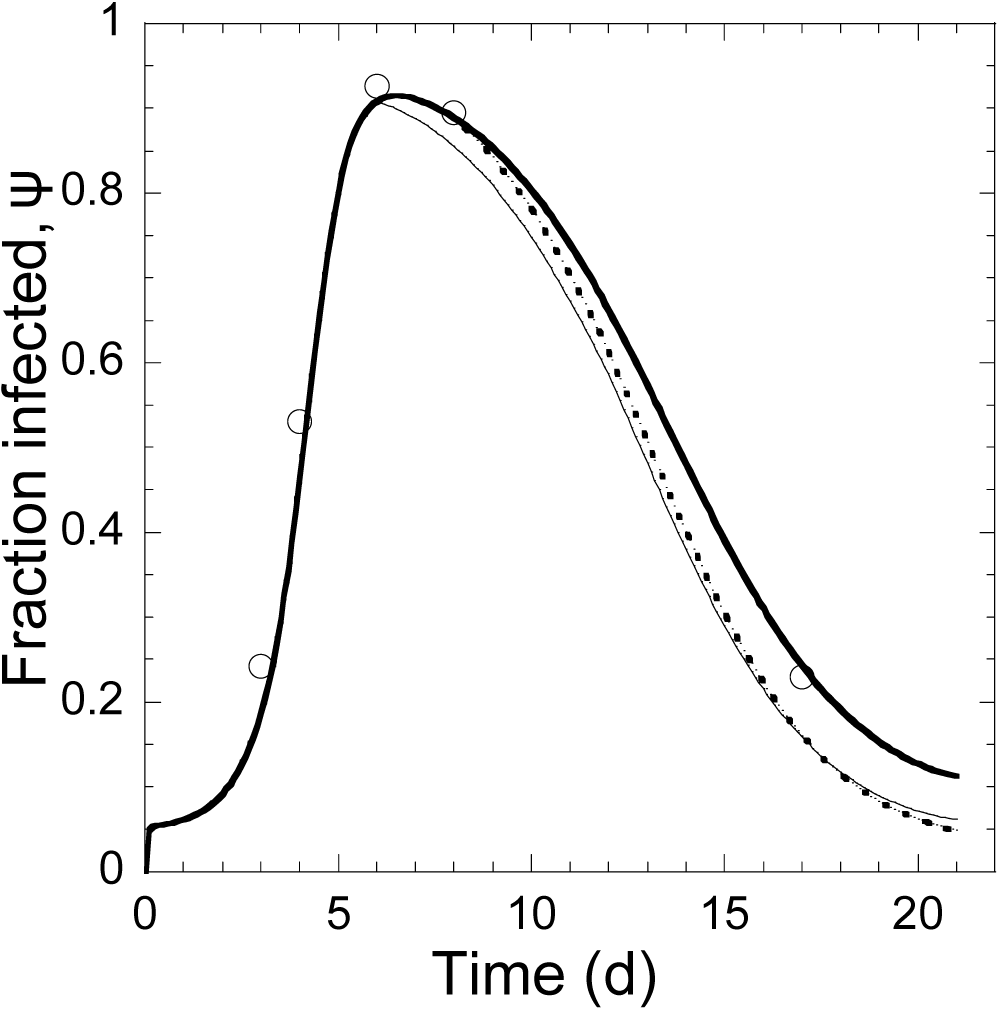

Supplement: Figure S7 — Influence of splitting of cell culture at confluence. Model predictions of the fraction of cells infected without splitting (thick line), splitting at day 6 and day 12 (thin line), and splitting at day 8 and day 12 (dashed line) after the onset of infection compared with the data in Fig. 3D (symbols). Parameters used are the same as in Fig. 3D. (TIF) [file pcbi.1002307.s007.tif]

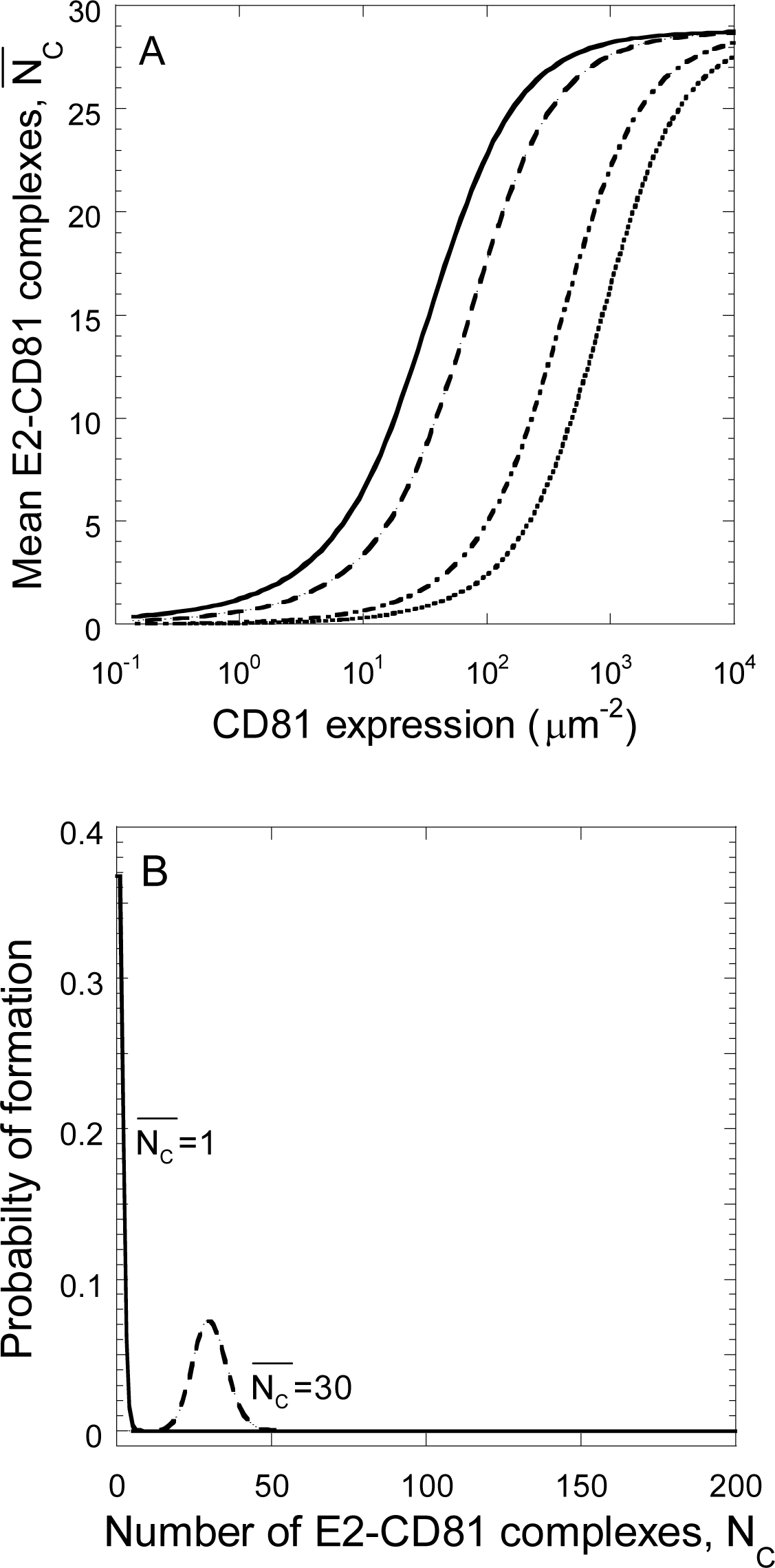

Supplement: Figure S8 — Mean number and probability of formation of E2-CD81 complexes. (A) Model predictions of the mean number of E2-CD81 complexes formed, , as a function of CD81 expression for = 1.7×10^−5^ M (solid line), 3.3×10^−5^ M (dashed line), 1.7×10^−4^ M (dashed-dotted line), and 3.3×10^−4^ M (dotted line). (B) Model predictions of the Poisson probability of forming E2-CD81 complexes (Eq. (5)) when (solid line) and (dashed line). (TIF) [file pcbi.1002307.s008.tif]

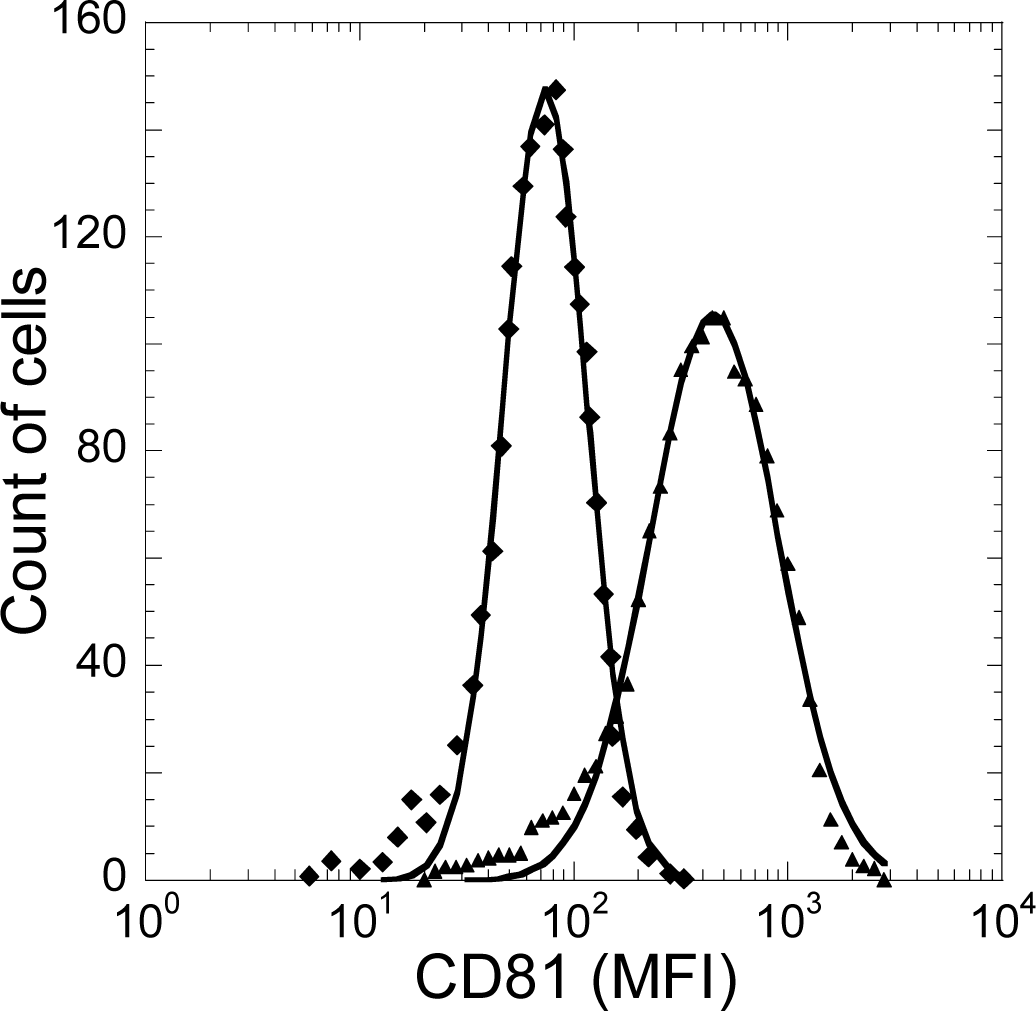

Supplement: Figure S9 — Conversion of fluorescence intensity to CD81 expression. Measured distributions of the CD81 expression level on Huh-7.5 (silRR) cells digitized from Zhang et al. (2004) J Virol 78:1448–1445 (diamonds) and Huh-7.5 cells from Koutsoudakis et al. (2007) J Virol 81:588–598 (triangles). Lines are best-fits of the log-normal distribution, , to the data. The best-fit parameter values (95% CI) are = 4.29 (4.27–4.31) and = 0.45 (0.43–0.47) for the data of Zhang et al. and = 6.1 (6.07–6.12) and = 0.7 (0.67–0.72) for the data of Koutsoudakis et al. (TIF) [file pcbi.1002307.s009.tif]
