# Supplementary material for: Mathematical Model of Viral Kinetics In Vitro Estimates the Number of E2-CD81 Complexes Necessary for Hepatitis C Virus Entry
Source: PLoS Comput Biol. 2011 Dec 8;7(12):e1002307. doi: 10.1371/journal.pcbi.1002307 (PMC3234214; doi:10.1371/journal.pcbi.1002307)
Supplement: Table S1 — Estimates of model parameters obtained from fits of model predictions to the data in Figs. 3B and S4A. 95% confidence intervals are indicated in brackets. (DOC) [file pcbi.1002307.s010.doc]

**Table S1.** Estimates of model parameters obtained from fits of model predictions to the data in Figs. 3B and S4A. 95% confidence intervals are indicated in brackets.

| $K_{D}$ [M] | *β* [ml•(ffu•d)^-1^] | *ω* [ffu•ml^-1^] | *δ* [d^-1^] | $N_{C}^{T}$ |
| --- | --- | --- | --- | --- |
| 1.7×10^-5^ | 1.1 (0.6-1.7)×10^-4^ | 0.13 (0.06-0.2) | 1.1 (0.4-1.8)×10^-2^ | 8.9 (5.3-12.5) |
| 3.3×10^-5^ | 1.1 (0.6-1.7)×10^-4^ | 0.13 (0.06-0.2) | 1.1 (0.4-1.8)×10^-2^ | 5.9 (3.5-8.2) |
| 1.7×10^-4^ | 1.2 (0.6-1.8)×10^-4^ | 0.12 (0.06-0.19) | 1.1 (0.4-1.8)×10^-2^ | 2.5 (1.7-3.3) |
| 3.3×10^-4^ | 1.3 (0.7-1.9)×10^-4^ | 0.12 (0.06-0.19) | 1.1 (0.4-1.8)×10^-2^ | 1.9 (1.3-2.4) |
