# Supplementary material for: Mathematical Model of Viral Kinetics In Vitro Estimates the Number of E2-CD81 Complexes Necessary for Hepatitis C Virus Entry
Source: PLoS Comput Biol. 2011 Dec 8;7(12):e1002307. doi: 10.1371/journal.pcbi.1002307 (PMC3234214; doi:10.1371/journal.pcbi.1002307)
Supplement: Table S2 — Estimates of model parameters obtained from fits of model predictions to the data in Figs. 3C and S4B. 95% confidence intervals are indicated in brackets. (DOC) [file pcbi.1002307.s011.doc]

**Table S2.** Estimates of model parameters obtained from fits of model predictions to the data in Figs. 3C and S4B. 95% confidence intervals are indicated in brackets.

| $K_{D}$ [M] | *β* [(TCID_50_•d)^-1^] | *p* [TCID_50_•d^-1^] | $N_{C}^{T}$ |
| --- | --- | --- | --- |
| 1.7×10^-5^ | 0.9 (0.7-1.1)×10^-4^ | 1.6 (1.0-2.2) | 7.8 (7.2-8.3) |
| 3.3×10^-5^ | 1 (0.8-1.2)×10^-4^ | 1.5 (1.0-2.1) | 4.8 (4.5-5.2) |
| 1.7×10^-4^ | 1.1 (0.9-1.3)×10^-4^ | 1.4 (0.9-1.9) | 1.8 (1.7-1.9) |
| 3.3×10^-4^ | 1.2 (1.0-1.4)×10^-4^ | 1.3 (0.9-1.8) | 1.33 (1.26-1.4) |
